# Supplementary figures and images for: Comparing local ancestry inference models in populations of two- and three-way admixture
Source: PeerJ. 2020 Oct 2;8:e10090. doi: 10.7717/peerj.10090 (PMC7537619; doi:10.7717/peerj.10090)

AFA\_two\_way haplotype ancestry distribution

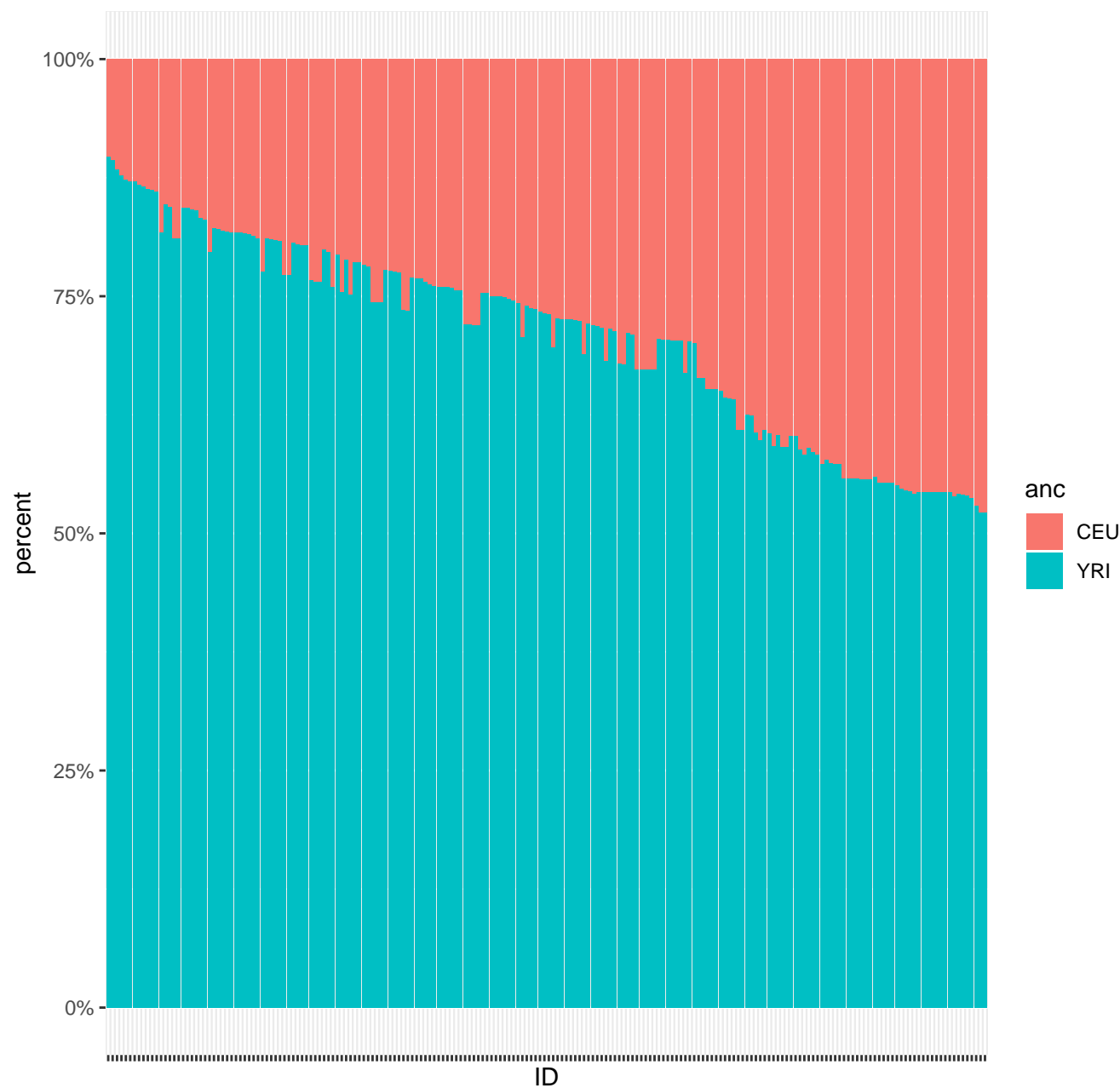

Supplement: Supplemental Information 1 — Each bar is an individual with their percentages of global ancestries represented per haplotype. [file peerj-08-10090-s001.pdf]

# HIS haplotype ancestry distribution

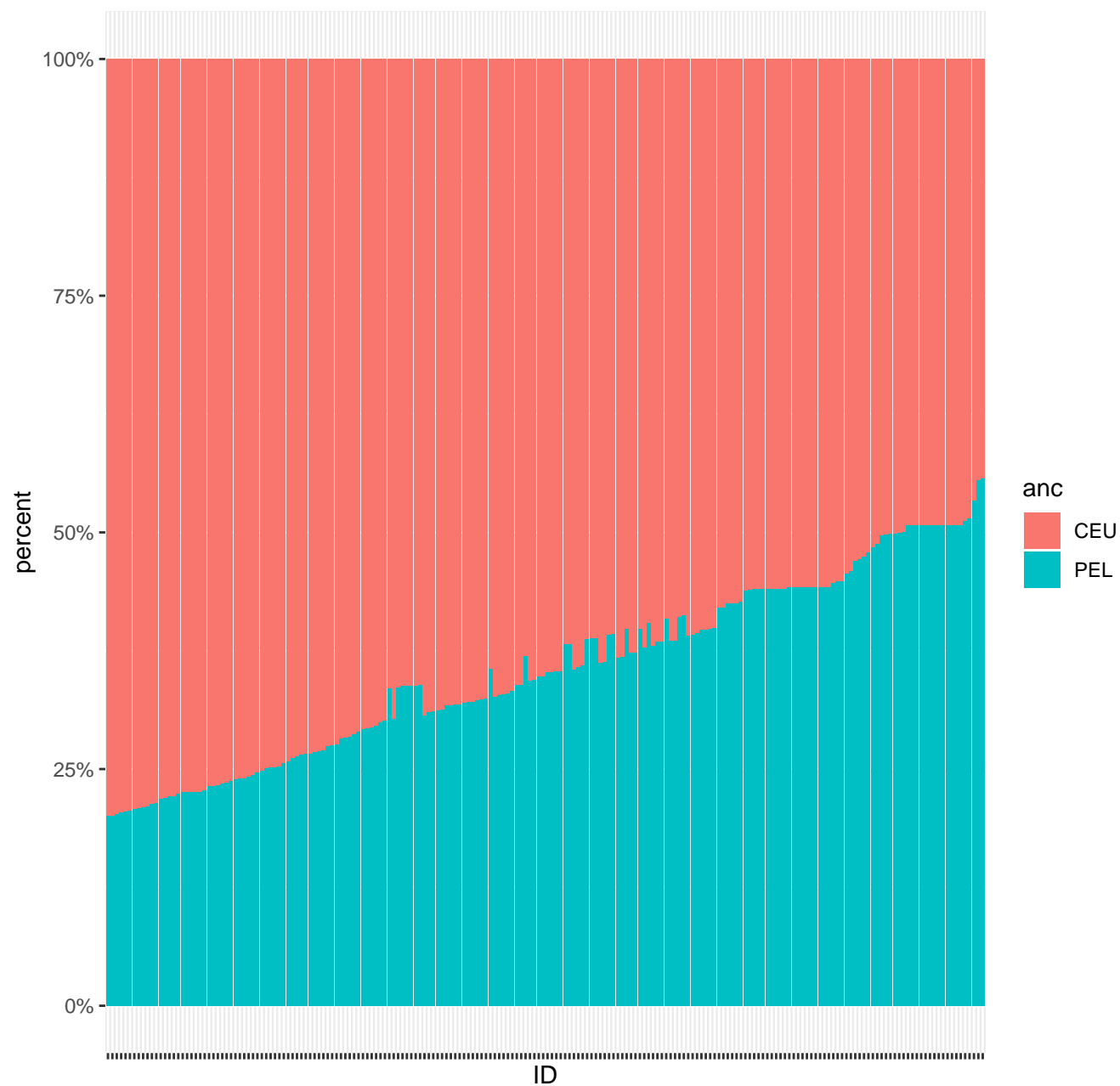

Supplement: Supplemental Information 2 — Each bar is an individual with their percentages of global ancestries represented per haplotype. [file peerj-08-10090-s002.pdf]

# 3WAY haplotype ancestry distribution

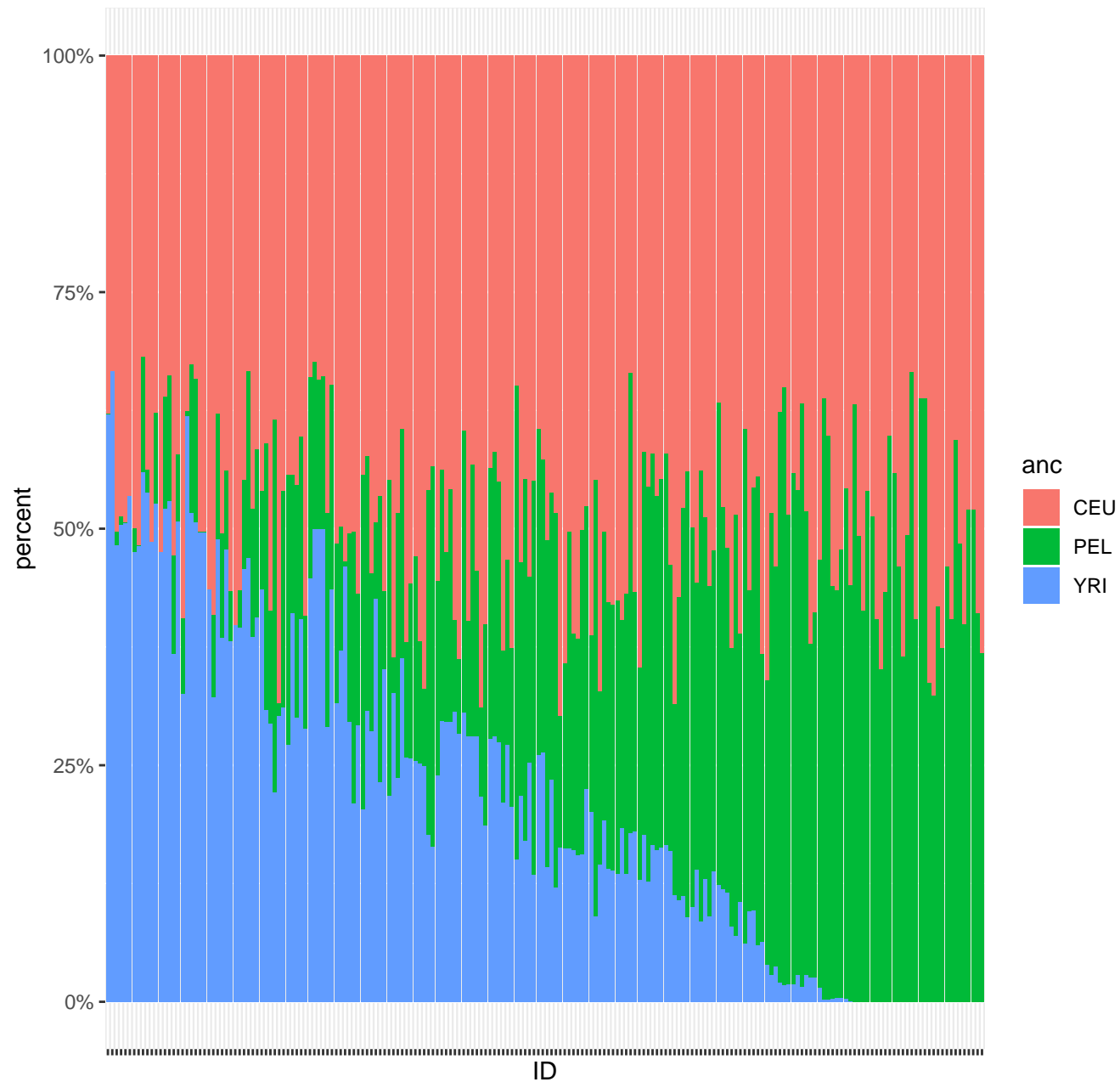

Supplement: Supplemental Information 3 — Each bar is an individual with their percentages of global ancestries represented per haplotype. [file peerj-08-10090-s003.pdf]

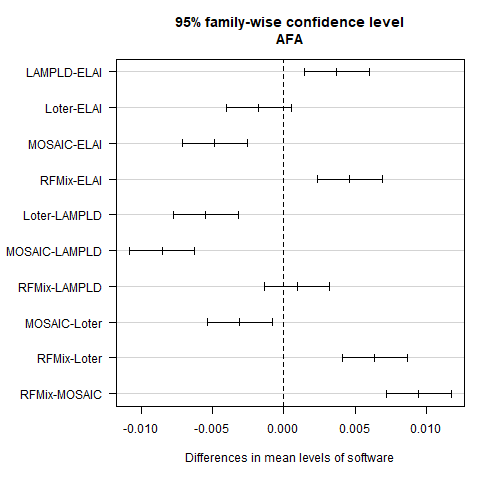

Supplement: Supplemental Information 4 — 95% family confidence intervals for Tukey’s test when running each software on a two way AFA admixed population. We find significant differences between the following pairs: RFMix & Loter (p = 4.0e − 13), RFMix & ELAI (p = 3.7e − 7), RFMix & MOSAIC (p < 2.14e − 14), LAMP-LD & Loter (p = 1.0e − 9), LAMP-LD & ELAI (p = 1.0e − 4), LAMP-LD & MOSAIC (p < 2.14e − 14), ELAI & MOSAIC (p = 1.0e − 7), and Loter & MOSAIC (p = 2.3e − 3). All other pairs were found to not have significantly different means [file peerj-08-10090-s004.png]

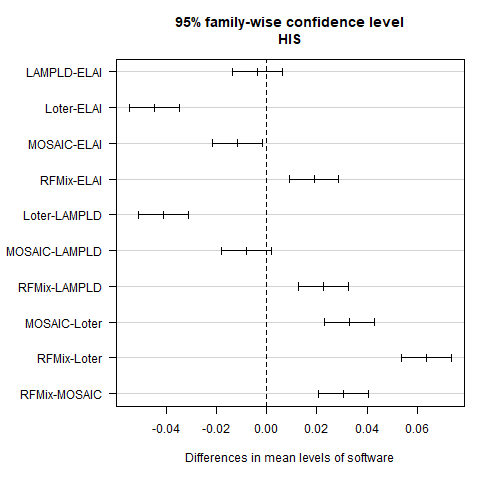

Supplement: Supplemental Information 5 — 95% family confidence intervals for Tukey’s test when running each software on a two way HIS admixed population. We find significant differences between the following pairs: RFMix & LAMP-LD (p = 8.0e − 9), RFMix & Loter (p < 2.14e − 14), RFMix & ELAI (p = 2.6e − 6), RFMix & MOSAIC (p < 2.14e − 14), ELAI & Loter (p < 2.14e − 14). ELAI & MOSAIC (p = 1.2e − 2). MOSAIC & Loter (p < 2.14e − 14), and LAMP-LD & Loter (p < 2.14e − 14). All other pairs were not found significantly different. [file peerj-08-10090-s005.png]

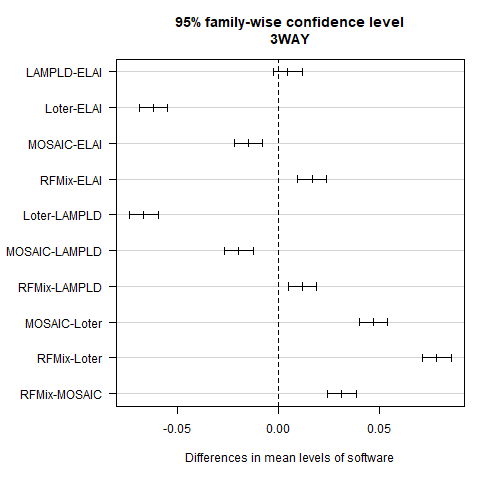

Supplement: Supplemental Information 6 — 95% family confidence intervals for Tukey’s test when running each software on three way admixed population. We find significant differences between the following pairs: RFMix & LAMP-LD (p = 4.6e − 5), RFMix & Loter (p < 2.14e − 14), RFMix & MOSAIC (p < 2.14e − 14), RFMix & ELAI (p = 1.8e − 9), LAMP-LD & Loter (p < 2.14e − 14), LAMP-LD & MOSAIC (p = 1.2e − 12), ELAI & Loter (p < 2.14e − 14), ELAI & MOSAIC (p = 1.8e − 7), and MOSAIC & Loter (p < 2.14e − 14). All other pairs were not found significantly different. [file peerj-08-10090-s006.png]
